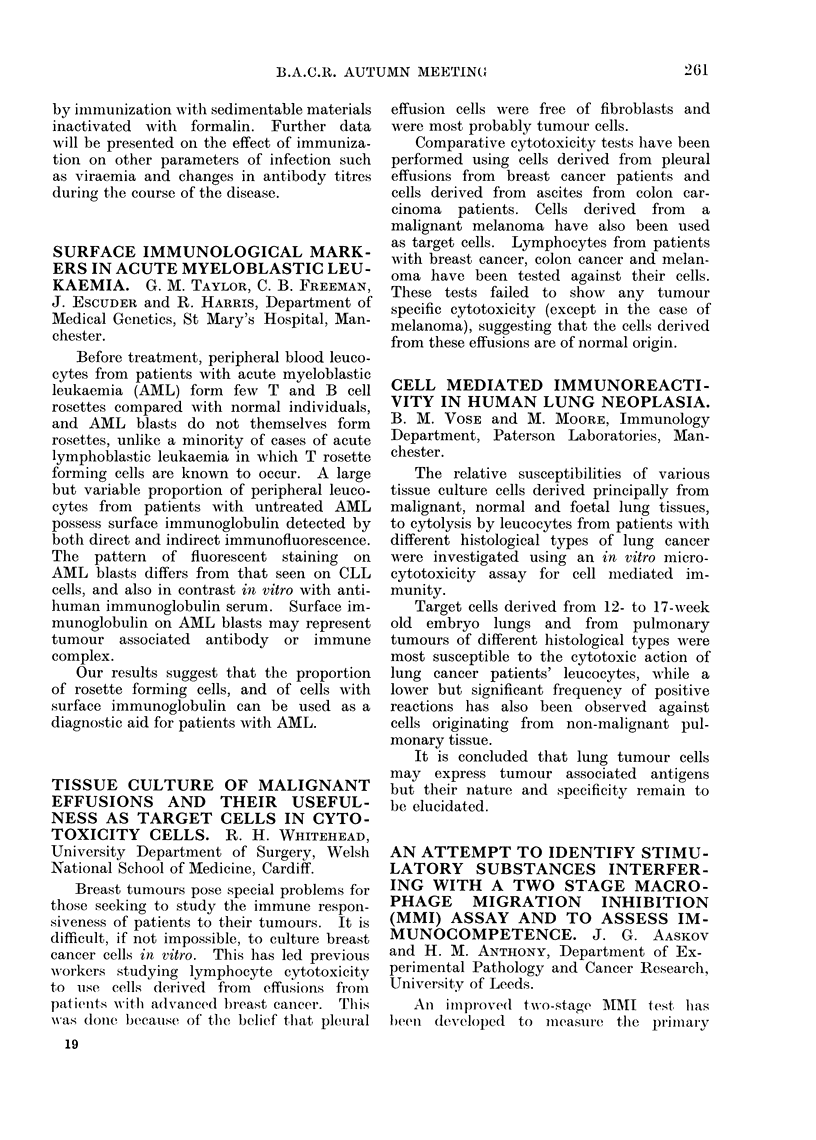# Proceedings: Tissue culture of malignant effusions and their usefulness as target cells in cytotoxicity cells.

**DOI:** 10.1038/bjc.1975.41

**Published:** 1975-02

**Authors:** R. H. Whitehead


**CELL MEDIATED IMMUNOREACTIVITY IN HUMAN LUNG NEOPLASIA.** B. M. VOSE and M. MOORE, Immunology Department, Paterson Laboratories, Manchester.

The relative susceptibilities of various tissue culture cells derived principally from malignant, normal and foetal lung tissues, to cytolysis by leucocytes from patients with different histological types of lung cancer were investigated using an *in vitro* microcytotoxicity assay for cell mediated immunity.

Target cells derived from 12- to 17-week old embryo lungs and from pulmonary tumours of different histological types were most susceptible to the cytotoxic action of lung cancer patients' leucocytes, while a lower but significant frequency of positive reactions has also been observed against cells originating from non-malignant pulmonary tissue.

It is concluded that lung tumour cells may express tumour associated antigens but their nature and specificity remain to be elucidated.